# Family Studies for Classification of Variants of Uncertain Classification: Current Laboratory Clinical Practice and a New Web-Based Educational Tool

**DOI:** 10.1007/s10897-016-9993-2

**Published:** 2016-07-16

**Authors:** Lauren T. Garrett, Nathan Hickman, Angela Jacobson, Robin L. Bennett, Laura M. Amendola, Elisabeth A. Rosenthal, Brian H. Shirts

**Affiliations:** 1 Department of Laboratory Medicine, University of Washington, Rm NW120, Box 357110, 1959 NE Pacific Street, Seattle, WA 98195 USA; 2 Department of Medicine, Division of Medical Genetics, University of Washington, Seattle, WA USA

**Keywords:** Variant of Uncertain Clinical Significance, VOUS, Genetics education, online patient education, Genetic counseling, Family segregation, co-segregation, family studies

## Abstract

Multi-gene cancer panels often identify variants of uncertain clinical significance (VUS) that pose a challenge to health care providers in managing a patient’s cancer risk. Family segregation analysis can yield powerful data to re-classify a VUS (as either benign or pathogenic). However, financial and personnel resources to coordinate these studies are limited. In an informal assessment we found that family studies for variant classification are done by most clinical genetics laboratories that offer hereditary cancer panel testing. The process for family studies differs substantially across laboratories. One near universal limitation is that families usually have too few individuals for an informative co-segregation analysis. A unique and potential resource-saving approach is to engage patients and their families in expanding their own pedigrees for segregation analysis of their VUS. We describe a novel public educational tool (FindMyVariant.org) designed to inform patients and genetic counselors about strategies to improve the probability of variant classification using familial segregation. While the web tool is designed to be useful for any gene, the project was primarily focused on VUS’s returned in cancer risk genes. FindMyVariant.org is a resource for genetic providers to offer motivated families who are willing to gather information about their family relationships and history. Working alongside clinical or research genetic laboratories, the information they collect may help reclassify their VUS using segregation analysis.

## Introduction

Panel gene sequencing has become an increasingly important strategy for evaluating inherited disease risk. Gene panels are now available for the work-up of conditions such as aortopathy, arrhythmia, cancer syndromes, cardiomyopathy, epilepsy, immunodeficiency, mitochondrial disorders, and X-linked intellectual disability (Chambers et al. 2016; Falk et al. 2012; Green et al. 2015; Hunter et al. 2014; Pritchard et al. 2014; Walsh et al. 2011). Expanded gene panels are more sensitive than single gene testing, and are often more cost effective than sequential testing, leading to additional diagnostic and prevention opportunities (Gallego et al. 2015). However, these panels have the caveat of also identifying rare variants of uncertain clinical significance (VUS) in a higher proportion of patients compared to single gene testing alone (Cragun et al. 2014; Kurian et al. 2014; Maron et al. 2012; Maxwell et al. 2014; Tung et al. 2014). A VUS typically has some characteristics or associated data indicating the variant may be deleterious, but not enough information to definitively classify it as disease causing, or pathogenic. Millions of such variants are present at low frequencies in the population (Lek et al. 2015).

The finding of a VUS can be problematic for patients and clinicians working in a clinical genetics setting (Culver et al. 2013; O’Neill et al. 2006; van Dijk et al. 2006; Vos et al. 2008). Although definitive re-classification of a VUS as pathogenic or benign may eventually occur, the timeline is typically many years, and may be indefinite for rare VUS, especially if the disease is uncommon (Lindor et al. 2013; Murray et al. 2011; O’Neill et al. 2009). In-silico or functional research studies usually cannot resolve the clinical significance of a variant (Katsonis et al. 2014; Lindor et al. 2012; Moghadasi et al. 2013; Schulz et al. 2015). While family segregation studies have the potential to yield powerful data to classify variants, many challenges emerge that limit this potential. The resources needed to identify distant family members and build the large pedigrees necessary to have enough information for informative segregation analysis are inadequate. (Goldgar et al. 2008; Mohammadi et al. 2009). While research studies dedicated to investigating rare variants in families could impact individual families, current research resources and funding are limited and unlikely to have an influential impact on a large number of families.

Most clinical genetics laboratories that offer multigene cancer panel testing do family studies for variant classification; however, practices and policies vary. Recognizing the need to assess the current landscape of family studies across clinical labs, we performed an informal assessment of 12 clinical laboratories located in the United States that offer cancer multigene panel testing using Next Generation Sequencing to determine their current VUS classification activities and procedures.

A major limitation to family studies for VUS reclassification is that the size of the family reported to genetics providers in clinical visits often includes only three generations of relatives and is too small to enable VUS classification (Eggington et al. 2013; Shirts et al. 2013). A majority of patients will have sufficient relatives to reclassify VUS within their first and second cousins (B.H. Shirts and Rosenthal 2015). However, explanation of the details of familial co-segregation studies may be beyond the scope of what could be expected of a genetics provider in routine clinical care.

In response to needs of patients with VUS reports and genetics providers who may be interested in family studies to classify VUS, we developed a novel public educational tool designed to inform patients and genetic counselors about strategies to improve the probability of VUS re-classification using familial segregation. The website will also be a resource for patients, genetic counselors, and other health professionals seeking clinical laboratories and research studies that perform family analysis for VUS classification. This web resource can be found at FindMyVariant.org.

## Assessment of Genetics Laboratories Offering Family Analysis Services for VUS Classification

We contacted 12 commercial clinical genetics laboratories by telephone and email and asked about current VUS re-classification activities, policies, and procedures between July and November 2015. Our questions were designed to include any VUS re-classification services as follow up on VUS findings *in any tested gene* where a VUS has been identified. Although these services benefit families that are able to reclassify their VUS, as accurate classification enables appropriate clinical care, (Murray et al. 2011), there is no established standard of care for VUS re-classification using family studies. Our goal was not to formally compare laboratories across specific metrics, but rather, to learn the current landscape of VUS re-classification practices.

We initially asked laboratories several broad questions: Is there a family studies program in place? What are the criteria and/or policy for eligibility? And what is the process for enrollment and sample collection? Based on responses we asked for clarification. We re-contacted several laboratories for clarification and to gather additional information. Representatives of each laboratory were given the opportunity to review final content for accuracy, although not all laboratories responded to our queries. Information about Myriad Genetics was gathered from the “MyVision” Variant Classification Program website. In addition to the laboratories listed in Table 1, we contacted Quest and Prevention Genetics, but these laboratories had no description of family studies on their respective web sites and did not respond to our phone queries. Because of the lack of community standards for VUS classification activities and the wide variety of practices, our assessment was not designed as a formal survey.

**Table 1:** Informal Assessment of genetics laboratories offering family analysis services for VUS classification

Lab Name and Family Studies Program*	Is there a Family Studies Program? What is a brief description?	What is the cost to patients and/or relatives?	How do you select the families for follow/up? Is every patient who is given a VUS in a cancer gene offered participation in family studies?	How long have you offered Family Studies?	What is the application process?
Ambry Genetics “Family Studies Program” http://www.ambrygen.com/family-studies-program	Yes. The cases approved for family studies are those that will be most informative in the variant classification process. The decision to approve or deny family studies occurs in the context of the particular variant and family; therefore, all cases are encouraged to apply (with the exception of the moderate penetrance genes listed below).	No charge if accepted into family studies program	It is an application process. Certain genes are excluded from the family studies program: At this point in time, patient’s whose results consist of only one VUS in the following genes are not eligible for Ambry’s Family Study Program: ATM, BARD1, BRIP1, CHEK2, MRE11A, NBN, RAD50, RAD51C, and RAD51D. When applications are received for patients with one VUS in these genes, the provider will be informed about the Inherited Breast and Ovarian Cancer Study at Mayo Clinic. Providers may refer their patients to this study directly without applying to Ambry’s Family Study Program. Individuals with two VUS in the same gene from this list are still eligible to apply to the Family Studies Program for the purpose of determining phase (cis/trans).	4 years or so but probably longer	There is a note put in the report if eligible for family studies with contact information on how to apply. Download forms and follow application process on website; it’s a patient application process.
ARUP “FAMS” program	Yes. A family studies program is in place via the University of Utah IRB approved FAMS research study. Every VUS report has a note to please contact a GC to assess possibility of family studies. Once appropriate relatives are identified, consents and kits are sent to everyone.	No charge through a research study	Application process. No real exclusionary /inclusionary criteria. No genes excluded from family studies research.	At least 10 years	Every report of a VUS has an addendum that asks to please contact a GC at ARUP to ask about feasibility of family studies
Baylor	There is not necessarily a “family studies” program in place, rather, individual cases reviewed on a case-by-case basis.	No charge for VUS in appropriate relatives if meets criteria	Case by case basis to see if appropriate. No set exclusion/inclusion criteria, cases are reviewed and deemed appropriate as they come in	At least 10 years, ever since cancer testing offered	Statement put in report if patient good candidate.
Counsyl	Yes. Counsyl has been doing BRCA1/2 since 2013 and recently launched cancer panels in June 2015. There has been a family studies process in place at the lab.	No charge for eligible relatives	Patients/providers who get a VUS result are asked for a copy of a 3-generation pedigree. It is reviewed and if eligible GC is contacted. At this point, no gene exclusions for family studies, if gene is offer it on Counsyl’s panels. There is a “get counsyl” option which features on demand genetic counseling and coordinating. This can be helpful for coordinating family studies, shipping kits and arranging blood draws for other relatives.	For BRCA1/2 since 2013. Most VUS to date have been in these genes, recently launched panels.	Patients/providers who get a VUS result are asked for a copy of a 3-generation pedigree. It is reviewed and if eligible, GC is contacted. Counsyl gives “opt -out” option for VUS to provider so not all providers receive VUS results.
Fulgent	Yes. Fulgent Diagnostics offers no-charge testing for qualifying relatives where a VUS has been reported by Fulgent.	No charge for eligible relatives	GC or care provider reaches out to Fulgent and they will work with patient/family on case-by-case basis. No set inclusion/exclusion criteria per say.	Since late 2013, since launching cancer genes	GC must contact Fulgent with information about the family (who is affected, and who is available for testing) to see if patient qualifies. Family study availability is not noted on test results.
GeneDX “Variant Testing Program”	Yes. There is family studies/variant testing program in place.	No charge for eligible relatives	There are no set inclusion or exclusion criteria. Reviewed on case-by-case process. Any gene they offer can potentially be worked up in family studies.	Since August 2013, since launching cancer genes	Provider gets VUS test result and it is up to provider to contact GeneDx if interested in Family Studies for variant reclassification. Provider can submit pedigree and clinical information, GeneDx will let provider know if family is eligible for testing and if so, which individuals qualify for testing.
Invitae “VUS Resolution Program” https://www.invitae.com/en/vus-resolution	Yes. In order to help resolve variants of uncertain significance (VUS), Invitae offers follow-up testing at no additional charge to select relatives of patients previously tested at Invitae. Variant reclassification relies on information gathered from multiple families. Participation may not result in an immediate reclassification, but can provide information that contributes to this process. If a variant is reclassified, Invitae will issue amended reports with the new interpretation to all individuals who have the variant. Invitae supports sharing our variant interpretations, and makes de-identified variant interpretations publicly available to the research and medical communities via the Clinvar and Clinvitae databases.	No charge for qualifying families. For Families that do not qualify for VUS resolution the cost is $200 per individual per gene in which a VUS was found.	Not all variants can be resolved with this kind of analysis, and not all relatives can provide informative data. Decisions about program eligibility are based on:The family structure, disease status, and clinical presentation of available individuals. The type of sequence change in question. The gene in which the VUS was identified. The inheritance pattern(s) and penetrance of the disease(s) associated with the gene. Some genes are not eligible. If eligible the original clinical report will include a statement recommending consideration of VUS resolution.	About 5 years	Statement put in report if patient good candidate for family studies. Patient application process is all online. Patients make an account that pulls in all patients tested information and builds online pedigree.
Myriad “MyVision” Variant Classification Program	Segregation analysis is one of several variant classification techniques used.	No charge for select patients and relatives	Myriad Genetic Laboratories scientists will review the family history and determine if family testing could provide data useful for reclassification	Over 10 years.	Most patients receiving variant result will get a variant information sheet, and an invitation to participate in Variant Classification Program. Provider sends in clinical history form and pedigree. If accepted, Myriad will send invitation and kit to provider to pass on to patients
Pathway Genomics “Family Studies Program**”**	Yes. Pathway Genomics offers a complimentary Familial Studies Program to help understand the significance of these genetic changes, and how the patient and relatives may be affected. Not all patients will qualify for complimentary testing.	No charge for patients and relatives	Any report where a VUS is detected will include an application for the clinician and patient to complete. Once familial application is received, it is reviewed within two to three weeks to determine program eligibility. For eligible families instructions will be sent to the ordering clinician that indicate which relatives are authorized for testing. Additional relatives who decide to participate will receive a complimentary laboratory report for the specific change variant in question as well as an interpretation of the current clinical implication.	No information provided.	If a VUS is detected, the report includes family studies application. Applications are reviewed by Familial Studies Program.
University of Washington http://depts.washington.edu/labweb/Divisions/MolDiag/MolDiagGen/	Yes. Families are reviewed on a case-by-case basis during multiple-medical-director case review. Families may be eligible for family studies through the University of Washington Department of Laboratory Medicine or on a research basis through research laboratories.	No charge for select patients and relatives	Director and laboratory GC review families. Availability of family analysis for clinical follow-up may be communicated by phone or email to providers. Ordering providers may also contact the laboratory to query about eligibility. Families are evaluated to determine if available relatives are likely to provide sufficient evidence to reclassify the VUS.	4 years	Provider must contact laboratory to discuss

*Of the twelve commercial clinical labs we surveyed, two labs, Prevention Genetics and Quest, did not respond to our inquiry

### Assessment Results

There are similarities and differences across the labs (See Table 1). Several commercial genetics laboratories (e.g., Ambry, GeneDx, Invitae) have family studies programs in place with well-developed inclusion/exclusion criteria and/or policies. For example, one lab excludes certain genes from their family studies program, such as low-moderate penetrance cancer genes like *ATM, BARD1, BRIP1, CHEK2, MRE11A, NBN, RAD50, RAD51C,* and *RAD51D*. Other laboratories tend to review family studies candidates on a case-by-case basis (e.g., ARUP laboratories, GeneDx, the University of Washington). Nearly all laboratories offer no laboratory fee for familial single-site testing for relatives that would inform segregation analysis for at least a subset of genes and carefully selected families. All laboratories that offer any form of family studies reported that the program began the same time they started offering clinical cancer gene testing. When asked how many families have been eligible for family studies and what the reclassification “success” rate was, each laboratory reported no mechanism for keeping track of how many patients were eligible, offered participation, or ultimately how many variants were reclassified due to successful co-segregation analysis via family studies. One interviewee explained that although there were many different ways and reasons a VUS could be reclassified, the reason why it was being reclassified, such as family study information, wasn’t being tracked.

In general, offering family studies was a common but not necessarily uniform practice for these clinical laboratories that perform genetic testing for hereditary cancer risk. Some of the most significant differences between laboratories are in the process for family studies. Some clinical laboratories work with patients and genetic counselors encouraging individuals to reach out to known family members and identify additional relatives in order to reclassify variants. For most clinical laboratories, however, family studies were only offered in selected situations where VUS reclassification is most likely to be possible or only for selected genes. Some laboratories offer family segregation studies to any interested family on a fee-for-service basis.

## Motivation to Develop General Online Resources for Family Based VUS Classification

As can be seen in Table 1, many clinical genetics laboratories currently offer family studies programs. These programs are diverse in how they operate and many place the onus on genetics providers to order testing and to help patients identify relatives and obtain biological specimens. Neither genetic counseling time nor the laboratory resources used for family study activities are reimbursed by insurance companies. Limited genetics provider resources and laboratory opportunities make it challenging for many willing patients to participate in successful VUS reclassification efforts.

While advertising the availability of family testing services, clinical laboratories simultaneously have mechanisms to control and restrict patient access to family segregation studies. Commercial laboratories may feel obliged to offer family based VUS reclassification services in limited instances because it is expected by genetics providers; however, there is little incentive to assist patients in contacting the large number of relatives necessary to reclassify VUS or to provide broad access to education about family studies for VUS classification as testing large numbers of relatives can be costly for laboratories. More importantly, the time and effort needed to work with patients and their relatives to build the 4–5 generation pedigrees that are usually necessary to classify VUS would be administratively challenging and prohibitively expensive.

A potential solution is to engage patients and their relatives in expanding their own pedigree. Although there are limited provider and laboratory resources for personally working with and educating patients about pedigree building for family studies, online genealogy resources and social networking may facilitate patients identifying and contacting their relatives themselves with minimal personal guidance. This observation sparked our interest in developing an educational resource for patient-driven family history building for the purpose of VUS classification.

## FindMyVariant.org, a New Resource for VUS Family Studies

We have developed a website that contains a patient-driven VUS reclassification toolkit. The mains goals of the toolkit are to: help individuals and families understand medical uncertainty about rare variants; educate individuals and families about ways to find more information about the variants that are unique to their family; provide resources for individuals who want to connect with their families to increase understanding about their genetic variants, and finally, provide resources for individuals who want to connect with clinical laboratories or research studies to learn more about their VUS.

The toolkit walks individuals step-by-step through the process of gathering the family specific segregation information required to reclassify any VUS that has been identified in clinical testing. This toolkit explains the variant reclassification process, teaches patients how to identify relatives who may also carry the VUS, (i.e. informative relatives), and provides aids for contacting relatives and describing the need to involve them in VUS classification. We sought insight from clinical genetic counselors, molecular genetic pathologists, bioethicists, and clinical geneticists to develop content for the website, and we worked alongside an experienced web developer to design the website in a patient- and user-friendly manner.

**Fig. 1:**
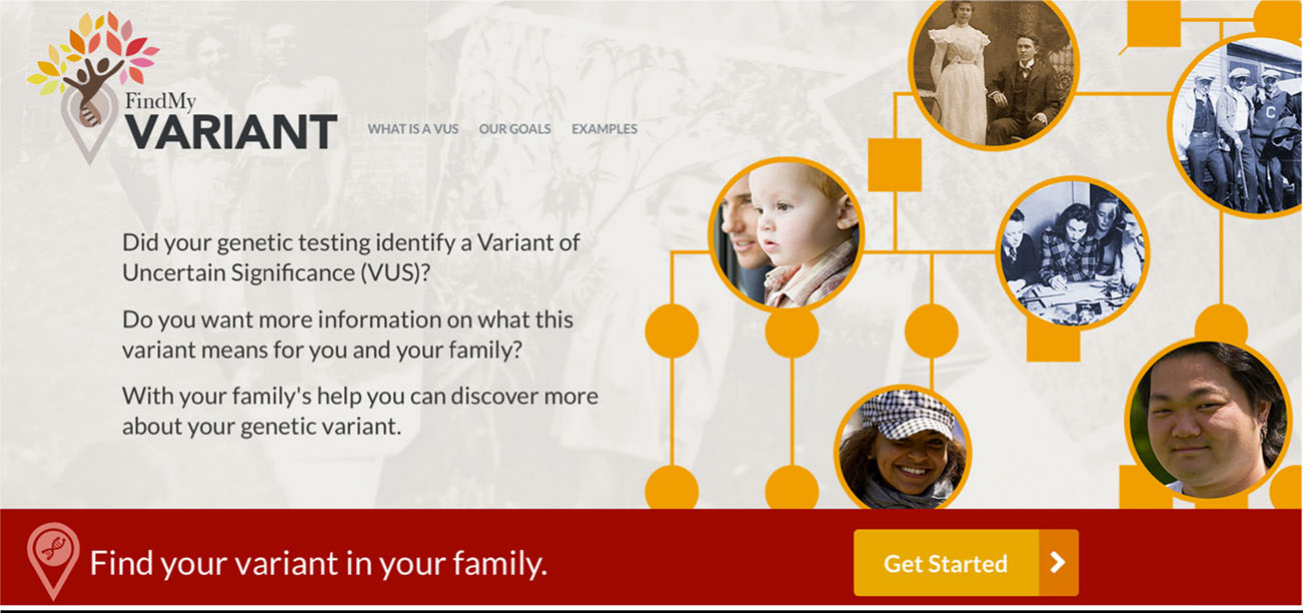
Screenshot of the home page for FindMyVariant.org, a public website. Patients can click on the “get started” prompt and will be introduced to a number of different modules (designed in order, as seen in Table 3) to walk them through the process of gathering family information to help them classify their variant

**Fig. 2:**
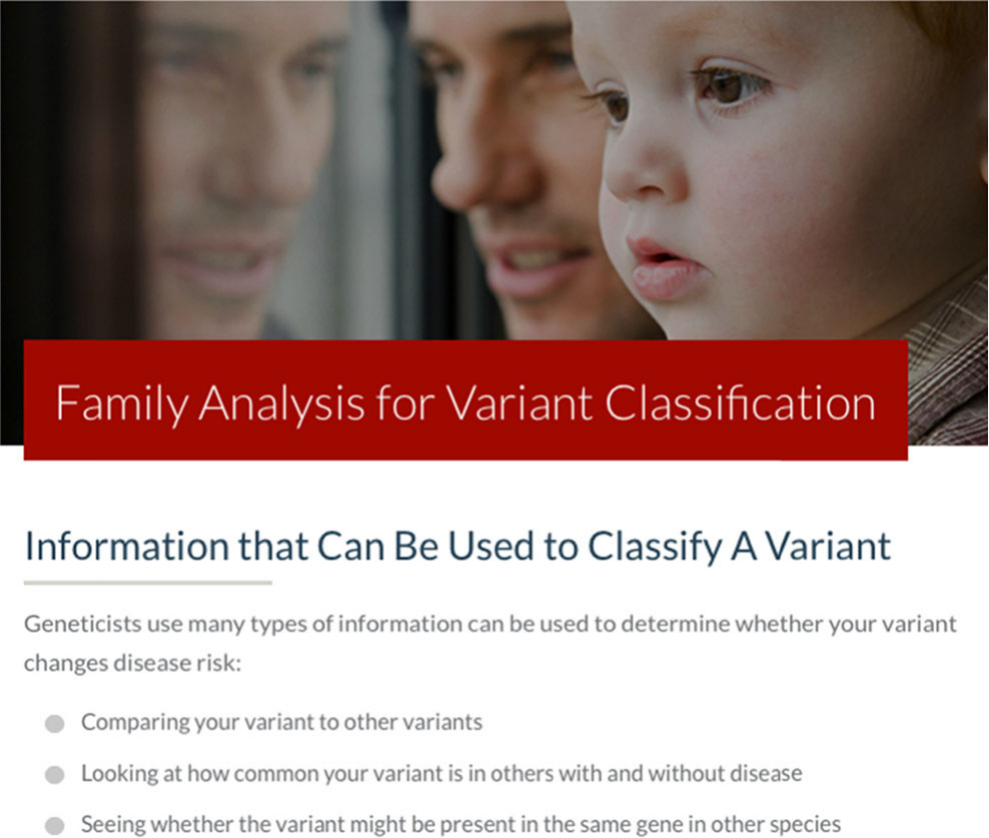
Screenshot with an example of an educational module found at FindMyVariant.org. This module walks a patient through the basics of using family information (i.e. via family studies) for family co-segregation analysis, evidence standards for variant classification, and classifying variant as either benign or pathogenic. It includes patient-friendly information and examples

**Fig. 3:**
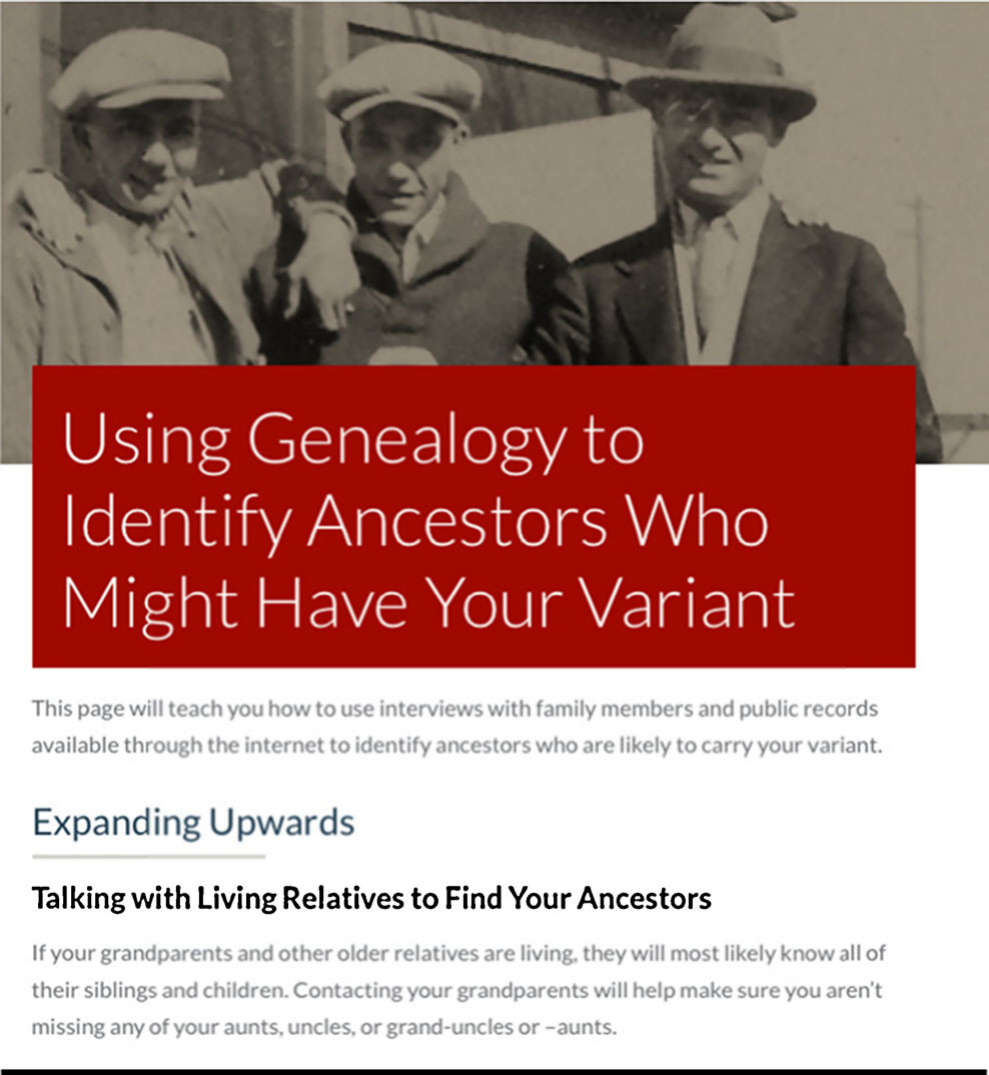
Screenshot with an example of another educational module found at FindMyVariant.org. This module walks a patient through the process of using online genealogy and social networking tools and resources to find and connect with relatives

**Fig. 4:**
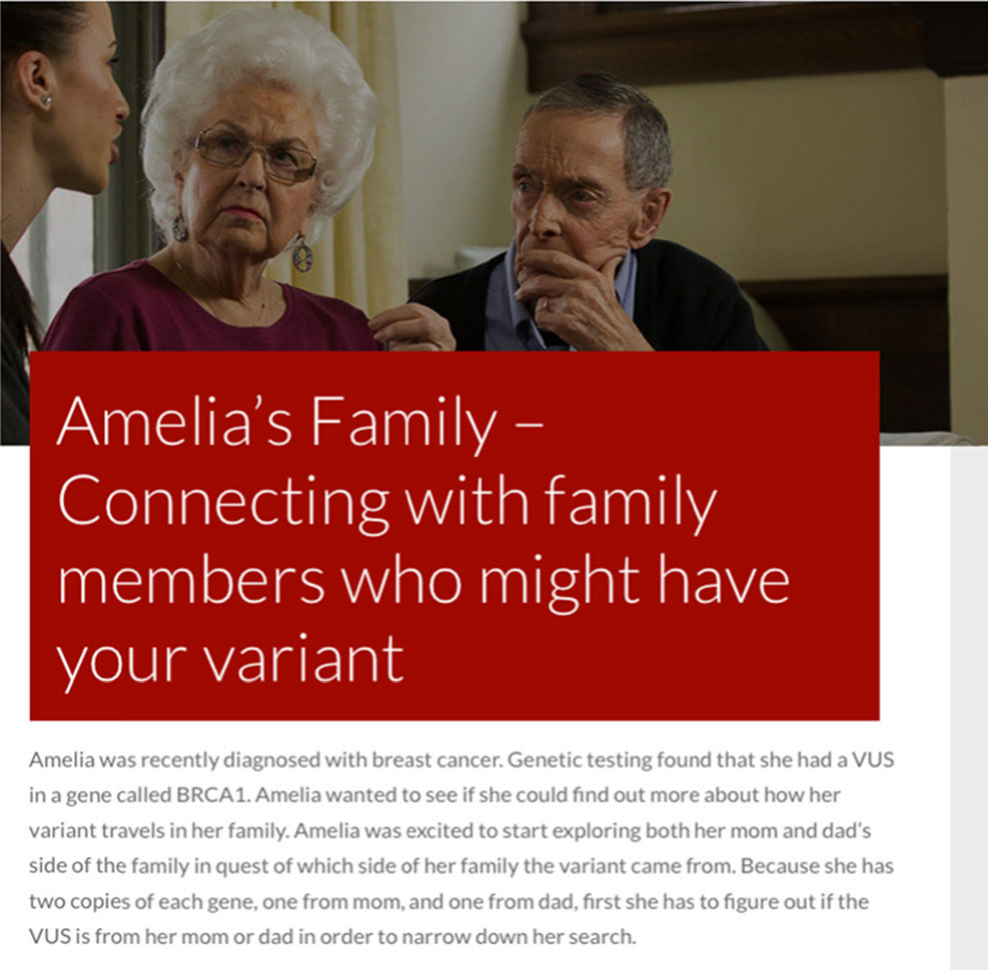
Screenshot illustrating one of two “family examples” on the website. Amelia is a patient who is eager to classify her BRCA1 VUS; this example demonstrates Amelia’s journey using different modules to help her connect with relatives and gather the necessary information need to ultimately reclassify her VUS to a pathogenic variant, conferring cancer risks for both her and her relatives also carrying the BRCA1 variant

The content of the website is designed and organized around eight modules or “steps” that a patient may encounter when gathering family medical and variant data (Table 2). The modules walk the patient through each step and include graphics and examples. Screenshots of the home page and of different modules are presented in Figs. 1, 2, 3 and 4. One module lists several online genealogy and social networking tools available to facilitate patients identifying and contacting their relatives, and describes how these might be used in the context of family history building for variant classification (see Fig. 3). In addition to this content, the website contains two examples that describe the experience of hypothetical individuals going through the process of attempting to reclassify the familial VUS following the tools offered in the website (see Fig. 4).

**Table 2:** Outline of online web modules for classifying a Variant

Module	Section	Content/Purpose
What Does VUS mean	What is a Genetic Variant	Explains variation in human disease and the different ways they are classified
	Genetic Variants & Disease	Variants can be associated with increased or decreased risk for disease
	Understanding Rare Variants	Most rare variants are benign, and certain rare variants can be unique in families
	Medical Care and VUS	Guidelines suggest not to alter medical management given a VUS, but rather, consider family history and other risk factors when assessing risk.
	Reclassifying VUS	Sufficient evidence may determine if a VUS does or does not cause disease in the family and reclassification of the VUS to benign or likely pathogenic can occur.
Co-Segregation Analysis for Variant Classification	Information that can be used to classify a variant	This information includes, comparing variant to other variants, looking at how often this variant occurs in individuals with disease, comparing this variant across species
	Basics of using family information for co-segregation analysis:	Describes methods and likelihood ratios used when performing family studies to determine if a variant is associated with disease
	Evidence standards for variant classification	Reviews different levels of classification assigned to variants ranging from benign to pathogenic.
	Classifying a pathogenic variant	Provides description and illustration of a pedigree for a pathogenic variant at different sizes/stages of analysis
	Classifying a benign variant	Provides description and illustration of a pedigree for a benign variant at different sizes/stages of analysis
	Using other information to help classify a variant	How geneticists can compile many sources of information to determine a likelihood ration that a variant is traveling with disease in a family.
Talking with your immediate family about your variant	Start with what you already know	Instructs individuals to start by recording pertinent personal information
	Organize information about your immediate family	Teaches how to add known family history information about close relatives
	Add what you know about your extended family	Describes adding information about health and disease in extended family members
	Talking with your family	Instruct how to be sensitive to family members while communicating with them.
	Contact your living parents and siblings	Explains how to collect, record, and confirm basic facts about close relatives.
Using genealogy to identify ancestors who might have your variant	Talking with living relatives to find your ancestors	Teaches how to gather information from living relatives about deceased ancestors.
	Using online genealogy tools to find your ancestors	Lists several genealogy resources including major family history websites with a very brief description of how to use these resources.
	Obtaining tumors from deceased ancestors for testing	Introduces the potential option of getting tissue from pathology samples from deceased ancestors and briefly describes this process.
Finding and Connecting with distant relatives that might have your variant	Start with the people you know	Explains that the best way to find distant relatives is by starting with people that you already know
	Talking with living relatives to find descendants of your ancestors	Describes how to talk to known relatives about descendants of a common ancestor, including how to ask for contact information of distant relatives.
	Using online social networking sites to find descendants of your ancestors	Provides examples of how to use social networking sits, like Facebook, to identify distant relatives.
	Don’t forget low-tech	Lists several public search engines used for finding information about relatives and a variety of people search tools.
Laboratory Testing and asking relatives about participation in VUS classification	Finding a clinical Laboratory to Test Samples and Classify Your Variant	Lists family study resources offered by several clinical laboratories in the United States.
	Finding a Research Laboratory to Test Samples and Classify Your Variant	Lists contact information for research studies performing family studies for VUS reclassification.
	Asking Family Members to Help You Classify Your VUS	Different clinical laboratories and research studies have different processes for sample submission. This module instructs participants to contact the testing laboratory directly to find details about how relatives can submit samples and offers tips for approaching family.
	Example Scripts to Ask a Family Member to Help Classify a Variant	Provides text examples of how to ask a family member to donate a sample to help classify a VUS.
Genetics Education	What is a gene?	Lists websites that provides explanations and resources for genetics learning tools
	What is a variant?	Lists websites that explain normal variation across humans and provides links learning tools
	Where do our genes come from?	Lists websites that explain basic concepts of heredity and de novo mutations
	How does heredity work?	Lists websites that describe inheritance patterns in families and individuals
	What is a mutation?	Lists websites that describe what changes in DNA are and how they happen.

“Amelia’s family” is an example of a woman with breast cancer who has a VUS in *BRCA1*. It includes descriptions of Amelia talking to her family, trying to obtain tumor tissue from a deceased ancestor, and using social media to find distant relatives. Ultimately, her VUS is tracking with cancer in her family and can be reclassified as pathogenic, causing increased cancer risk in relatives who carry the same variant. Similarly, “Charlie’s family” is an example of a man with colon cancer and a VUS in *APC* that might be associated with attenuated familial adenomatous polyposis. This example includes descriptions of a patient talking to his family, uncovering information about his great-grandparents, and discovering a branch of his family tree of which he was not aware. The example concludes with Charlie learning that the genetic variant he carries does not cause cancer risk and is reclassified as likely benign. The goal of these examples is to mimic as realistically as possible some of the smaller successes and challenges a motivated patient could expect to come across when attempting to gather all the information that is needed to successfully classify a variant using co-segregation analysis in their own family. Further, they demonstrate the two different outcomes that can be expected upon successful completion of family studies: in Amelia’s family, the VUS was reclassified to pathogenic, while in Charlie’s family, his VUS was reclassified as likely benign (See Fig. 4, Table 2).

One specific challenge to patients with VUS that are interested in family studies to learn more about their VUS, is the difficulty in obtaining laboratory services to genotype and classify their variants. As illustrated in Table 1, each laboratory has different processes and criteria for eligibility. Laboratories also have different variant classification criteria and may draw different conclusions from the same family data (Yorczyk et al. 2015). Although, the “Laboratory Testing and Asking Relatives About Participation in VUS Classification” module of the web tool lists clinical laboratories and research investigations involved in family studies and provides links to relevant information, we could not outline next steps for interacting with clinical and research laboratories because there is no uniform process. This is a stage in family studies for variant classification where the patient will necessarily need to contact the testing laboratory for guidance about how to proceed.

## Discussion

The issue of large numbers of patients with variants of uncertain significance is already a problem for medical genetics practice (Culver et al. 2013; O’Neill et al. 2009; Lindor et al. 2013), and may become greater as large-scale sequencing enters clinical care. It is estimated that each individual has hundreds of rare, family-specific variants that might be classified as VUS. Interpreting VUS in clinical genetics creates challenges for health care providers, and their patients. Despite the potential of family studies for VUS reclassification, the resources available and offered to patients are limited.

We surveyed 12 laboratories offering NGS panels for cancer genes that perform family studies for genetic variant classification and provide a summary of eligibility criteria and processes where these details are defined by the testing laboratories reviewed. While not designed to be a formal survey of laboratory practices, we hope this information is a useful resource for genetic counselors and other health professionals as they help patients found to carry a VUS to understand potential next steps for resolving its clinical significance.

While family studies have the potential to yield powerful data to reclassify variants, the time and effort required to build pedigrees adequately for variant classification limit this potential. We developed an educational website containing a patient-friendly toolkit to help aid patients in working with research and clinical laboratories to reclassify their own VUS. The modules were developed to walk patients step by step through the process and address some of the realistic challenges that might come up along the way. Recognizing that each patient and family has it’s own complexity and challenges, we offered two examples of patients (Fig. 4: Amelia and Charlie, see screenshot) from families suspicious for hereditary breast and hereditary colorectal cancers, respectively, as representative examples.

While the website was developed with the above benefits in mind, we recognize that this is a first attempt at designing a patient-friendly toolkit for variant reclassification and consequently, has limitations. At this time, the toolkit is designed for English speaking patients. Further, while we have attempted to build resources suitable for most patient education levels, we realize that some of the concepts and content may be beyond the comprehension level of some patients or their relatives. Because the website conveys complex terms in a simplified level, it may be easier for patients of all educational levels to use. Each patient’s family is different and subsequently, the number of individuals needed to reclassify a VUS will be different for each family, and based on the gene and rarity of the disease. There are potential barriers to patients having access to the website, either because their ordering health care provider is not aware that it exists, or they or their relatives may not have internet or computer resources necessary to access the website. Privacy and data security concerns prevented us from including interactive pedigree building at this time; we are investigating this as a possibility in future versions.

As more patients use the website, other challenges that we did not anticipate are likely to be identified. Although reclassification is theoretically possible for almost all families given enough effort in pedigree building, practicalities may prevent many individuals from being able to use family studies to reclassify their variants such as individuals who were adopted, families who used assisted reproductive technologies utilizing donor gametes, or individuals who have limited knowledge or contact with relatives. Further, it is possible that only highly motivated patients will be willing to make the effort to grow their family histories to the point where they are able to classify their variants. Each laboratory we interviewed uses different rubrics for identifying individuals that qualify for family studies and for reclassifying VUS through family studies. Laboratories will undoubtedly respond differently to patient efforts to grow their pedigrees for successful co-segregation analysis. There are still many unanswered questions about VUS classification. Few laboratories report keeping quality records of the success rate of family studies, so future studies will be needed to explore patient satisfaction with VUS classification activities and outcome metrics of these efforts.

## Conclusion

In the current paradigm of multi-gene panel testing, the return of a result with one or more VUS’s is a challenge faced by genetic and other health care providers and ultimately, their patients and families. Many laboratories are involved in conducting family studies for variant reclassification with diverse protocols and eligibility criteria.

Engaged patients who are empowered to overcome this barrier to VUS reclassification may make it possible for variant reclassification to shift from that of a research activity, to a clinical activity enabling individualized genomic medicine. In addition to enhancing understanding of rare variants encountered in clinical testing, this approach could provide a significant benefit to families faced with VUS findings. We have described a web tool that responds to this need, enabling patients to take charge of their genetic information in the age of genomic medicine.

## References

[CR1] Chambers, C., Jansen, L. A., & Dhamija, R. (2016). Review of commercially available epilepsy genetic panels. *Journal of Genetic Counseling, 25*(2), 213–217. doi:10.1007/s10897-015-9906-9. 10.1007/s10897-015-9906-9 26536886

[CR2] Cragun D, Radford C, Dolinsky JS, Caldwell M, Chao E, Pal T (2014). Panel-based testing for inherited colorectal cancer: a descriptive study of clinical testing performed by a US laboratory. Clinical Genetics.

[CR3] Culver, J., Brinkerhoff, C., Clague, J., Yang, K., Singh, K., Sand, S., & Weitzel, J. (2013). Variants of uncertain significance in BRCA testing: evaluation of surgical decisions, risk perception, and cancer distress. *Clinical Genetics, 17*(10), 12097. doi:10.1111/cge 10.1111/cge.12097 PMC3751990 23323793

[CR4] Eggington JM, Bowles KR, Moyes K, Manley S, Esterling L, Sizemore S, Wenstrup RJ (2013). A comprehensive laboratory-based program for classification of variants of uncertain significance in hereditary cancer genes. Clinical Genetics.

[CR5] Falk, M. J., Pierce, E. A., Consugar, M., Xie, M. H., Guadalupe, M., Hardy, O.,... Gai, X. (2012). Mitochondrial disease genetic diagnostics: optimized whole-exome analysis for all MitoCarta nuclear genes and the mitochondrial genome. *Discovery Medicine, 14*(79), 389–399. PMC3923327 23272691

[CR6] Gallego, C. J., Shirts, B. H., Bennette, C. S., Guzauskas, G., Amendola, L. M., Horike-Pyne, M.,. .. Veenstra, D. L. (2015). Next-generation sequencing panels for the diagnosis of colorectal cancer and polyposis syndromes: A Cost-Effectiveness Analysis. *Journal of Clinical Oncology, 33*(18), 2084–91. doi:10.1200/JCO.2014.59.3665. 10.1200/JCO.2014.59.3665 PMC4461806 25940718

[CR7] Goldgar DE, Easton DF, Byrnes GB, Spurdle AB, Iversen ES, Greenblatt MS (2008). Genetic evidence and integration of various data sources for classifying uncertain variants into a single model. Human Mutation.

[CR8] Green A, Green H, Rehnberg M, Svensson A, Gunnarsson C, Jonasson J (2015). Assessment of HaloPlex amplification for sequence capture and massively parallel sequencing of arrhythmogenic right ventricular cardiomyopathy-associated genes. The Journal of Molecular Diagnostics.

[CR9] Hunter AG, Graham JM, Neri G, Rogers RC, Stevenson RE, Turner G, Friez MJ (2014). The intellectual disabilities evaluation and advice system (IDEAS): outcome of the first 55 cases. American Journal of Medical Genetics. Part A.

[CR10] Katsonis P, Koire A, Wilson SJ, Hsu TK, Lua RC, Wilkins AD, Lichtarge O (2014). Single nucleotide variations: biological impact and theoretical interpretation. Protein Science.

[CR11] Kurian AW, Hare EE, Mills MA, Kingham KE, McPherson L, Whittemore AS, Ford JM (2014). Clinical evaluation of a multiple-Gene sequencing panel for hereditary cancer risk assessment. Journal of Clinical Oncology.

[CR12] Lek, M., Karczewski, K., Minikel, E., Samocha, K., Banks, E., Fennell, T.,. .. MacArthur, D. (2015). Analysis of protein-coding genetic variation in 60,706 humans. *Biorxiv*. doi:10.1101/030338 10.1038/nature19057 PMC5018207 27535533

[CR13] Lindor NM, Guidugli L, Wang X, Vallee MP, Monteiro AN, Tavtigian S, Couch FJ (2012). A review of a multifactorial probability-based model for classification of BRCA1 and BRCA2 variants of uncertain significance (VUS). Human Mutation.

[CR14] Lindor NM, Goldgar DE, Tavtigian SV, Plon SE, Couch FJ (2013). BRCA1/2 sequence variants of uncertain significance: a primer for providers to assist in discussions and in medical management. The Oncologist.

[CR15] Maron BJ, Maron MS, Semsarian C (2012). Genetics of hypertrophic cardiomyopathy after 20 years: clinical perspectives. Journal of the American College of Cardiology.

[CR16] Maxwell, K. N., Wubbenhorst, B., D’Andrea, K., Garman, B., Long, J. M., Powers, J.,. .. Nathanson, K. L. (2014). Prevalence of mutations in a panel of breast cancer susceptibility genes in BRCA1/2-negative patients with early-onset breast cancer. *Genetics in Medicine, 11*(10), 176. doi:10.1038/gim.2014 10.1038/gim.2014.176 PMC4465412 25503501

[CR17] Moghadasi S, Hofland N, Wouts JN, Hogervorst FB, Wijnen JT, Vreeswijk MP, van Asperen CJ (2013). Variants of uncertain significance in BRCA1 and BRCA2 assessment of in silico analysis and a proposal for communication in genetic counselling. Journal of Medical Genetics.

[CR18] Mohammadi, L., Vreeswijk, M. P., Oldenburg, R., van den Ouweland, A., Oosterwijk, J. C., van der Hout, A. H.,. .. van Houwelingen, H. C. (2009). A simple method for co-segregation analysis to evaluate the pathogenicity of unclassified variants; BRCA1 and BRCA2 as an example. *BMC Cancer, 9*(211). doi:10.1186/1471-2407-9-211. 10.1186/1471-2407-9-211 PMC2714556 19563646

[CR19] Murray, M. L., Cerrato, F., Bennett, R. L., & Jarvik, G. P. (2011). Follow-up of carriers of BRCA1 and BRCA2 variants of unknown significance: variant reclassification and surgical decisions. *Genetics in Medicine, 13*(12), 998–1005. doi:10.1097/GIM.0b013e318226fc15 10.1097/GIM.0b013e318226fc15 21811163

[CR20] O’Neill SC, DeMarco T, Peshkin BN, Rogers S, Rispoli J, Brown K, Schwartz MD (2006). Tolerance for uncertainty and perceived risk among women receiving uninformative BRCA1/2 test results. American Journal of Medical Genetics. Part C, Seminars in Medical Genetics.

[CR21] O’Neill SC, Rini C, Goldsmith RE, Valdimarsdottir H, Cohen LH, Schwartz MD (2009). Distress among women receiving uninformative BRCA1/2 results: 12-month outcomes. Psycho-Oncology.

[CR22] Pritchard CC, Salipante SJ, Koehler K, Smith C, Scroggins S, Wood B, Walsh T (2014). Validation and implementation of targeted capture and sequencing for the detection of actionable mutation, copy number variation, and gene rearrangement in clinical cancer specimens. The Journal of Molecular Diagnostics.

[CR23] Schulz, W. L., Tormey, C. A., & Torres, R. (2015). Computational approach to annotating variants of unknown significance in clinical next generation sequencing. *,Laboratoriums Medizin 46*(4), 285–289. doi:10.1309/LMWZH57BRWOPR5RQ 10.1309/LMWZH57BRWOPR5RQ 26489672

[CR24] Shirts, B. H., & Rosenthal, E. (2015, Oct 9, 2015). *Power of Single Extended Pedigrees to Classify Rare Variants of Uncertain Significance in BRCA1 and BRCA2.* Paper presented at the American Society of Human Genetics 65th Annual Meeting, Baltimore, MD.

[CR25] Shirts BH, Jacobson A, Jarvik GP, Browning BL (2013). Large numbers of individuals are required to classify and define risk for rare variants in known cancer risk genes. Genetics in Medicine.

[CR26] Tung, N., Battelli, C., Allen, B., Kaldate, R., Bhatnagar, S., Bowles, K.,. .. Hartman, A. R. (2014). Frequency of mutations in individuals with breast cancer referred for BRCA1 and BRCA2 testing using next-generation sequencing with a 25-gene panel. *Cancer, 3*(10), 29010. 10.1002/cncr.29010 25186627

[CR27] van Dijk S, Timmermans DR, Meijers-Heijboer H, Tibben A, van Asperen CJ, Otten W (2006). Clinical characteristics affect the impact of an uninformative DNA test result: the course of worry and distress experienced by women who apply for genetic testing for breast cancer. Journal of Clinical Oncology.

[CR28] Vos J, Otten W, van Asperen C, Jansen A, Menko F, Tibben A (2008). The counsellees’ view of an unclassified variant in BRCA1/2: recall, interpretation, and impact on life. Psychooncology..

[CR29] Walsh T, Casadei S, Lee MK, Pennil CC, Nord AS, Thornton AM, Swisher EM (2011). Mutations in 12 genes for inherited ovarian, fallopian tube, and peritoneal carcinoma identified by massively parallel sequencing. Proceedings of the National Academy of Sciences of the United States of America.

[CR30] Yorczyk A, Robinson LS, Ross TS (2015). Use of panel tests in place of single gene tests in the cancer genetics clinic. Clinical Genetics.

